# List of reviewers 2022

**DOI:** 10.1192/bjb.2023.18

**Published:** 2023-10

On behalf of the editorial board, the Editor-in-Chief would like to thank the following for their contribution as peer reviewers in the period from January 2022 – December 2022. Their anonymous work for the journal forms the foundation to its success, and is highly appreciated.

- Mohammed Abousaleh
- Alex Acosta-Armas
- Katherine Adlington
- Gwen Adshead
- Niruj Agrawal
- Jennifer Ahrens
- Neil Armstrong
- William Badenhorst
- Cate Bailey
- Chloe Beale
- Rahul Bhattacharya
- Vishal Bhavsar
- Jed Boardman
- Derek Bolton
- Helen Bruce
- William Burbridge-James
- Tom Burns
- Patricia Casey
- Oriana Chao
- Mike Crawford
- Ananta Dave
- Virginia Davies
- Lynne Drummond
- Sharif El-Leithy
- Carolina Estevao
- Waleed Fawzi
- Nicol Ferrier
- Paul French
- Linda Gask
- Clare Gerada
- Jaswant Guzder
- Lene Halstrup
- Catherine Hayhurst
- Jill Hemmington
- Claire Hilton
- Josanne Holloway
- Jeremy Holmes
- Ahmed Huda
- Peter Huxley
- Declan Hyland
- George Ikkos
- Clare Inkster
- Jeremy Isaacs
- Jeremy Johnson
- Edgar Jones
- Nikolina Jovanovic
- Simon Kirwin
- Harold Koenig
- Marie Kolkenbrock
- Michalis Kyratsous
- Andrew Larner
- Tennyson Lee
- William Lee
- Joanne Lloyd
- Kerry Louw
- Kate Lovett
- Jonny Martell
- Edel Mc Glanaghy
- Laura McWhirter
- Paravathy Mohands
- Akshay Nair
- Rajan Nathan
- Giles Newton-Howes
- Tesheen Noorani
- Aileen O'Brien
- Mary O'Kane
- Niall O'Kane
- Femi Oyebode
- Laura Panagi
- Nick Panay
- Reena Panchal
- A-La Park
- Carol Paton
- Vaios Peritogiannis
- John Peteet
- Rob Poole
- Sotiris Posporelis
- Claire Pouncey
- Stefan Priebe
- Denise Ratcliffe
- Keith Rix
- Frank Rohricht
- Alexander Ruck Keene
- Mark Salter
- Elizabeth Sampson
- Abdi Sanati
- Tim Segal
- Gertrude Seneviratne
- Swaran Singh
- Valentin Skryabin
- Soraia Sousa
- Anne Stewart
- Harriet Stewart
- Annie Swanepoel
- Tayyeb Tahir
- Bruce Tamilson
- Anna Taylor
- Panneerselvamm Theerthaana
- Derek Tracy
- Peter Tyrer
- Benjamin Underwood
- Chleo Van Velsen
- Indira Vinjamuri
- Birgit Vollm
- Sean White
- Owen Whooley
- Stephen Wilson
- Marisa Wray

